# Cervical Syphilitic Lymphadenitis in a 29-Year-Old Female: A Case Report

**DOI:** 10.7759/cureus.36065

**Published:** 2023-03-13

**Authors:** David D Bickford, Peter Johnson, Nupur Brahmbhatt, Steven Kroft

**Affiliations:** 1 Internal Medicine, Medical College of Wisconsin, Milwaukee, USA; 2 Pathology, Medical College of Wisconsin, Milwaukee, USA

**Keywords:** lymphadenitis, lymphoma, cervical lymphadenopathy, hiv, syphilis

## Abstract

Cervical lymphadenopathy is a common condition characterized by the enlargement of lymph nodes. It can have various causes, including infections, inflammatory conditions, and neoplastic processes. Syphilis, a sexually transmitted disease that progresses through multiple stages, can also be a rare cause of cervical lymphadenopathy, particularly in HIV-positive individuals. In this case report, we describe a patient presenting with throat pain, systemic symptoms, and cervical lymphadenopathy, initially clinically suggestive of lymphoma but ultimately determined to be caused by syphilis of unknown duration. This case highlights the importance of considering syphilis in the differential diagnosis of cervical lymphadenitis, particularly in patients with risk factors, such as intravenous drug use and HIV infection, and the need for a thorough evaluation of the patient's social and medical histories to diagnose and treat the condition accurately.

## Introduction

Cervical lymphadenopathy refers to the enlargement of lymph nodes in the cervical chain. The differential diagnosis in adults includes various potential causes, such as infections, inflammatory conditions like rheumatoid arthritis or sarcoidosis, and neoplastic processes, including lymphoma and metastasis from other cancers. However, infections are the most frequent cause of lymphadenopathy and can be due to various bacterial, viral, and fungal pathogens. Infectious culprits include streptococcal pharyngitis, cat scratch disease, mononucleosis, tuberculosis, and syphilis.

Syphilis is a sexually transmitted disease with progressive stages of infection [1]. Primary syphilis typically occurs two to six weeks post-infection, presenting with a single, painless genital ulcer (chancre) and inguinal lymphadenopathy [1]. Untreated cases may progress to secondary syphilis, presenting with rash, fever, generalized lymphadenopathy, mucosal patches, condyloma lata, and alopecia [1]. The number of primary and secondary syphilis cases in the United States has been rising, with the Centers for Disease Control and Prevention (CDC) reporting an increase of 71.4% since 2014 [1]. The increased infection rate was primarily among men, accounting for 87% of all syphilitic infections, and 42% of those patients also reported co-infection with human immunodeficiency virus (HIV) [1]. However, the increased rates of syphilis in the community also expose other vulnerable populations. A resurgence of syphilis is being reported in heterosexual women of childbearing age, especially those with histories of intravenous drug use or high-risk sexual behavior [1,2]. As of 2018, heterosexual women comprised 14% of total syphilis cases reported in the United States [1]. Reports of congenital syphilis have subsequently increased each year, with a 185% increase in 2018 relative to 2014 [1]. Syphilis acquired up to four years prior to childbirth, if left untreated, can cause congenital infection in over 80% of cases and lead to infant death or stillbirth in almost half [3].

Syphilitic lymphadenitis of the neck is a rare presentation of syphilis that can affect the lymph nodes in the cervical chain [4]. Co-infection with HIV and syphilis has been previously associated with atypical clinical manifestations and the course and severity of syphilitic infection in these patients can be more severe [5]. With prior data suggesting an increased risk of syphilis with co-existing HIV infection and intravenous drug use, alongside the risk of congenital syphilis occurring in women of childbearing age, renewed attention is needed to understand and manage syphilitic disease. Clinical diagnosis of syphilis can be missed or delayed due to varied features, incomplete clinical histories, and the reluctance of disclosure by patients for illicit or stigmatized activities. Additionally, as patterns of sexual activity and community risk change, patient populations and clinical presentation subsequently vary. Here, we report a case of throat pain and cervical lymphadenopathy with systemic systems, a constellation suggestive of lymphoma, ultimately discovered to be due to syphilitic lymphadenitis.

## Case presentation

Our patient was a 29-year-old Caucasian female admitted to the hospital following one week of unilateral, painful neck swelling, pain with swallowing, and left upper quadrant pain. Initial symptoms also included throat tightness, wheezing, hoarseness, ear pain, tinnitus, and dysphagia. She endorsed three months of fatigue, night sweats, and weight loss, which she attributed to drug use. She reported recent abdominal pain with constipation. The patient denied recent fevers, chills, shortness of breath, chest pain, palpitations, genitourinary symptoms, nausea, vomiting, or blood in the stool. She had no recent sick contacts, vision changes, arthralgias, body or genital lesions, pruritis, or body rashes.

**Figure 1:**
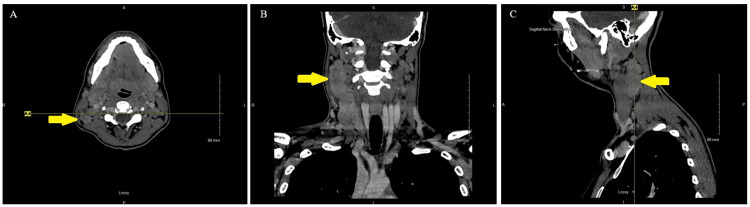
CT of the head and neck obtained at an outside hospital three days prior to admission Note: Shown are axial (Panel A), coronal (Panel B), and sagittal (Panel C) views of the cervical lymph mass. CT images were obtained with soft-tissue windows showing enlarged right level IIb/3 and 4a lymph nodes that extend craniocaudally subjacent to the sternocleidomastoid muscle. Most lymph nodes do not demonstrate normal fatty hilum. These findings increase concern for lymphoma over malignant lymphadenopathy or reactive adenopathy.

The patient had been evaluated for these symptoms at an outside hospital, and a computed tomography (CT) neck with contrast was performed. This image series found numerous enlarged lymph nodes in the neck, the right side greater than the left. The right level 2A lymph node measured 22 x 18 millimeters (mm) in the short axis, and the right level IIb/3 lymph node measured 17 x 27 mm in the short axis but extended at least 5 cm craniocaudally. The swollen lymph nodes did not demonstrate a normal fatty hilum (Figure 1). Additional non-enlarged confluent right level 4A lymph nodes were present, along with several supraclavicular lymph nodes. The basic metabolic panel (BMP) and complete blood count (CBC) were unremarkable. However, the Epstein-Barr virus (EBV) immunoglobulin G (IgG) antibody was elevated to 6.7 units per milliliter (U/ml). The outside hospital prescribed the patient cephalexin 500 milligrams (mg) twice daily and recommended transfer to our hospital for continued workup with malignancy concerns.

The patient’s medical history was significant for anxiety, asthma, and bipolar disorder. Current medications included an albuterol HFA inhaler for asthma, hydroxyzine for anxiety, and buprenorphine 4 mg with naloxone 1 mg sublingual film twice daily. The patient reported a one-year history of exclusively intranasal heroin use, though the physical exam revealed antecubital needle marks. The patient recently moved to a drug rehabilitation facility and never traveled outside the region. She reported homelessness for greater than a year and attended a shelter once, where she was sexually assaulted. She did not seek medical attention after the assault. She initially denied other recent sexual activity but, upon further questioning, reported she had engaged in sex work. She subsequently endorsed frequent high-risk sexual activity with multiple male partners with limited healthcare access. Additional family history included a mother and grandmother with lymphoma. Prior surgical history included a childhood tonsillectomy.

A physical exam revealed a well-appearing female in no acute distress. The cardiac exam showed a normal rate and regular rhythm with no murmurs, rubs, or gallops. Respiratory and abdominal exams revealed mild bilaterally wheezing in the upper lobes, significant left upper quadrant tenderness to mild and deep palpation, and mild splenomegaly. Pupils were equal, round, and reactive to light and accommodation, with anicteric sclera and clear conjunctivae. A thorough mouth and throat examination showed poor dentition without mucosal lesions. There was unilateral right-sided cervical lymphadenopathy with significant tenderness to palpation. The tender cervical node was indurated, non-fluctuant, and non-mobile, without overlying skin changes. Tenderness to palpation was also noted at the right axillary region and right supraclavicular node. There were no anogenital lesions or inguinal lymphadenopathy. Multiple healed lesions and scars were noted in the antecubital fossa.

**Figure 2:**
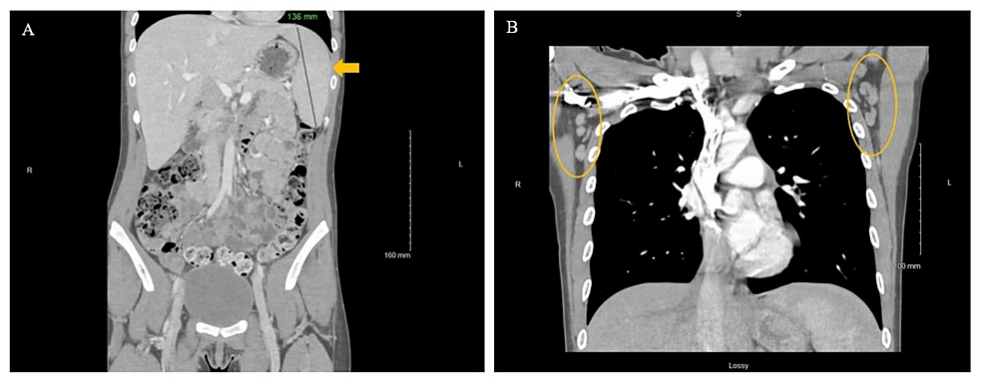
CT of the chest, abdomen, and pelvis Note: Shown are the coronal images of the abdomen (Panel A) obtained with soft tissue windows from CT performed with the administration of intravenous contrast material, showing mild splenomegaly, the spleen measuring 13.6 cm, without hepatomegaly. Coronal soft tissue CT imaging (Panel B) of axillary lymph nodes was unremarkable. No prior imaging was available for comparison. These findings are less consistent with suggested malignancy through the chest, abdomen, or pelvis. No other acute abnormalities were seen.

Pertinent laboratory values included mildly elevated aspartate and alanine aminotransferases of 45 units per liter (U/L) and 67 U/L, respectively, and an elevated C-reactive protein (CRP) of 2.2 milligrams per deciliter (mg/dl). The CBC and BMP remained unremarkable when compared to the outside hospital values. Other laboratory test results are shown in Table 1. The patient’s presentation and prior imaging findings were suggestive of reactive lymphadenitis secondary to infection from EBV or acute HIV etiology versus malignancy, favoring lymphoma. A CT chest, abdomen, and pelvis with contrast enhancement was conducted due to concern for malignancy and showed an enlarged spleen measuring 13.5 centimeters (cm). No lymphadenopathy or other discrete CT findings to suggest malignancy were seen throughout the chest, abdomen, or pelvis (Figure 2). The cephalexin started at the outside hospital was discontinued on the second day of treatment due to the low likelihood of bacterial infection.

**Table 1:** Summary of the patient’s initial laboratory data Note: RR = Reference range; AST = Aspartate aminotransferase; ALT = Alanine aminotransferase; Alk Phos = Alkaline phosphatase; LDH = Lactate dehydrogenase; CRP = C-reactive protein; ESR = Sedimentation rate; HIV = Human immunodeficiency virus; RPR = Rapid plasma reagin; IgG = Immunoglobulin G; IgM = Immunoglobulin M; Ab = Antibody; Hep = Hepatitis; NAAT = Nucleic acid amplification test; RNA = Ribonucleic acid; EBC = Epstein-Barr virus

Variable	Value	RR, Adults
AST	45 U/L	11 – 33 U/L
ALT	67 U/L	6 – 37 U/L
Alk Phos	91 U/L	35 – 104 U/L
Albumin	3.9 g/dl	3.8 – 5.0 g/dl
Protein, Total	7.0 g/dl	6.1 – 8.2 g/dl
Bilirubin, Total	0.3 mg/dl	0.2 – 1.2 mg/dl
LDH	192 U/L	135 – 214 U/L
CRP	2.20 mg/dl	< 0.50 mg/dl
ESR	14 mm/hr	0 – 28 mm/hr
HIV 1/2 Ab, Antigen	Reactive	Nonreactive
HIV-1 Ab, Differentiation	Reactive	Nonreactive
Absolute # T-Helper CD4+	437 cells/uL	410 – 1,540 cells/uL
RPR Screen	Reactive	Nonreactive
RPR Titer	1:8	None
Treponemal IgG + IgM Ab	Reactive	Nonreactive
Hep C NAAT Quantitative	Detected	Not Detected
Hep C RNA IU	71,200 IU/ml	Not Detected IU/ml
EBV Early Antigen Antibody IgG	112 U/ml	0.0 - 10.9 U/ml

The patient was scheduled for a cervical lymph node biopsy to exclude lymphoma. Concurrently, a broad infectious workup was performed for infection-related lymphadenopathy, including EBV titers, cytomegalovirus serology, and HIV testing. Additional testing included TB QuantiFERON, chlamydia trachomatis nucleic acid amplification test (NAAT), Neisseria gonorrhea NAAT, toxoplasmosis serology, hepatitis panel, Blastomyces antigen, histoplasmosis antigen, and rapid plasma reagin (RPR) with treponemal antibody testing. Results revealed elevated EBV IgG antibody of 112 U/ml compared to 6.7 U/L three days prior, HIV-1 antibody positivity with an absolute CD4+ cell count of 437 cells per microliter (uL), indicating new infection, and hepatitis C reactivity. The RPR screen was reactive with 1:8 titer, and treponemal IgG and IgM tests were reactive (Table 1). All other results were negative or unreactive. Upon further questioning, the patient did not endorse any ulcerative lesions in the anogenital region; however, she stated that under the influence of heroin, she may not have noticed a painless ulcer. Per CDC guidelines, diagnostic lumbar puncture was not performed due to low RPR titer and lack of neurological symptoms [6].

**Figure 3:**
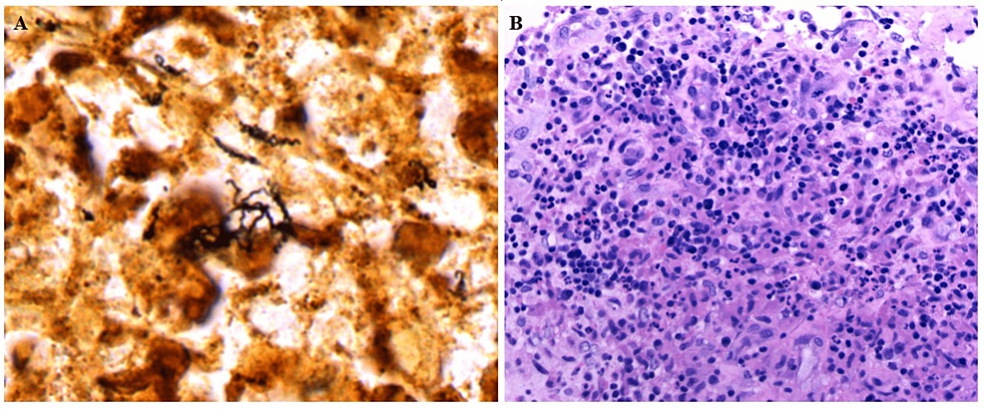
Bedside tissue biopsy specimens of the cervical lymph node chain Note: Showing a Warthin-Starry special stain (Panel A) of the right cervical lymph node biopsy specimen. The specimen shows lymphohistiocytic inflammation with spirochetes present, characteristic of *Treponema pallidum* infection. Panel B is a medium high-power hematoxylin and eosin (H&E) stain showing a mixed inflammatory infiltrate from the right cervical lymph node biopsy specimen.

The biopsy demonstrated foci of intact nodal tissue without evidence of malignancy alternating with fibrotic areas containing a mixed inflammatory infiltrate of histiocytes, plasma cells, and neutrophils. CD3 and CD20 staining revealed admixed small, mature T cells and B cells, respectively, while CD30 and CD15 failed to reveal evidence of Hodgkin cells. Warthin-Starry stain revealed spirochetes (Figure 3). Grocott methenamine silver, Gram, and acid-fast-bacilli special stains were all negative. Based on serology and biopsy results, the patient was diagnosed with syphilis. To determine our treatment plan, we had extensive conversations with the patient and our infectious disease specialists to elucidate symptom onset, however, without a known infection timeline, syphilis of unknown duration was the final diagnosis. She was treated with an initial dose of 2.4 million units of intramuscular penicillin G benzathine in the hospital. One day after her first penicillin G benzathine treatment, her neck pain and swelling began to decrease.

Her tenderness in the left upper quadrant, axilla, and supraclavicular regions also resolved. However, the patient did experience a brief instance of fevers, chills, and aches consistent with a Jarisch-Herxheimer reaction. The patient declined further intramuscular injections, citing pain and fear of large needles, and was prescribed a 28-day course of oral doxycycline 100 mg twice daily per 2021 CDC sexually transmitted infection (STI) treatment guidelines for syphilis of unknown duration [6], which she completed outpatient. In her outpatient infectious disease clinic, she was started on once-daily bictegravir/emtricitabine/tenofovir alafenamide for HIV infection. She received additional hepatitis C treatment with close clinical follow-up by an infectious disease specialist. Four months post-admission, repeat RPR was nonreactive, and CD4+ cell count showed 1026 cells/uL. Her cervical lymphadenopathy, left upper quadrant tenderness, and systemic symptoms had completely resolved. Her follow-up visit schedule was discussed, including outpatient visits every three months for a one-year duration, followed by a 24-month follow-up visit to ensure adequate serologic response and treatment success.

## Discussion

This case report describes a 29-year-old Caucasian female with a history of asthma, anxiety, intravenous drug use, and sex work, and a family history of lymphoma, presenting to the emergency department with rapidly progressive cervical lymphadenitis, splenomegaly with left upper quadrant tenderness, odynophagia, and systemic symptoms. The symptoms of axillary and supraclavicular lymph node tenderness, systemic symptoms, and positive family history of lymphoma initially suggested malignant etiology; however, serology results indicated a syphilitic origin with co-existing HIV-1 and hepatitis C infection.

The diagnosis for this patient was initially misled by her extensive family history of lymphoma and reluctance to disclose her history of sex work. On admission, she stated she had not had intercourse in three years and was unconcerned about STIs. She later admitted that she had been embarrassed to disclose her sexual history and was open to a full STI workup. HIV-infected patients are at increased risk for both Hodgkin’s and non-Hodgkin’s lymphomas, which can present in the cervical chain [7]. While lymph nodes' size and rapid growth were concerning for a rapidly aggressive lymphoma [8], tenderness to palpation and young age were more suggestive of an inflammatory cause of lymphadenopathy rather than neoplastic. Additionally, the patient’s CBC and CD4+ cell count was normal, making advanced lymphoma less likely, and definitive exclusion of the diagnosis of lymphoma was made by histological analysis of the cervical lymph node. At outpatient follow-up, the patient's cervical lymphadenopathy had resolved completely with treatment along with her systemic symptoms. With bictegravir/emtricitabine/tenofovir alafenamide initiation for her HIV infection, her risk for future HIV-associated lymphomas was also reduced [9], and the importance of continued antiretroviral therapy for risk reduction was discussed.

*Treponema pallidum*, the bacterium that causes syphilis, is transmitted through sexual contact or contact with infected bodily fluids, such as blood or saliva. The classic presentation of primary syphilis includes a painless ulcer at the site of infection, followed by a rash on the palms and soles of the feet, fever, and swollen lymph nodes [1]. Secondary syphilis manifests with additional symptoms, including mucosal lesions, alopecia, periostitis, hepatitis, and nephritis [10]. Syphilis can become asymptomatic in the latent phase, and relapses with recurrent infectious lesions have been reported during the early latent period in up to 24% of patients, potentially increasing infectivity [6,10]. Syphilitic lymphadenitis can occur during any stage of infection, causing lymph node swelling and tenderness alongside fever and fatigue. Syphilitic lymphadenitis typically manifests as painful cervical or inguinal masses, similar in appearance to inflammatory pseudotumors. In these cases, direct involvement of *Treponema pallidum* with the lymph node is typically described [11,12]. Hepatosplenomegaly can also be seen with secondary syphilis, and transaminitis with mildly elevated alkaline phosphatase has been reported [13].

Syphilis is treated by antibiotics, particularly penicillin, which is highly effective for all stages of syphilis, including congenital infection [14]. The standard treatment course includes one to three intramuscularly injected doses of 2.4 million units of long-acting penicillin G benzathine, depending on the stage of illness and severity of symptoms [15]. Due to a lack of follow-up, multiple intramuscular injections may be difficult for patients with low healthcare literacy, transportation issues, or drug relapse. Patients with HIV and primary or secondary syphilis should continue to be evaluated clinically and serologically for possible treatment failure at three, six, nine, 12, and 24 months after therapy, which can also increase the risk of loss to follow-up [6]. Consequently, up to 40% of adults who do not receive sufficient treatment can develop a tertiary disease with significant complications [16,17]. HIV-infected patients have been shown to respond less effectively serologically when compared to those without HIV infection, although penicillin treatment failure was very uncommon [18]. HIV infection increases the risk for neurosyphilis in untreated patients, typically accompanied by a serum RPR titer of 1:32 or higher, CD4+ count less than 350 cells/uL, and absence of HIV treatment, which were not observed in this case [19].

Secondary syphilis was our initial diagnosis for this patient. However, despite extensive conversations with the patient to determine symptom onset and timeline of infection, we ultimately had insufficient evidence to conclude onset prior to 12 months. Given the unclear symptom timeline, low nontreponemal titers of 1:8 (titers higher > 1:32 usually represent more acute infection), and extensive history with sex work, we determined, in collaboration with our infectious disease specialists, to treat this case as syphilis of unknown duration. This diagnosis and subsequent treatment strategy were based on the 2018 CDC sexually transmitted disease surveillance [1] and 2021 CDC STI treatment guidelines [6], which recommend that when faced with uncertainty, clinicians should act conservatively and treat syphilis of unknown duration the same as a latent syphilitic infection. Additionally, these sources recommend that if an alternative to penicillin is used, the only acceptable regimen for latent syphilis or syphilis of unknown duration is doxycycline (100 mg twice daily) or tetracycline (500 mg four times daily) for 28 days. The efficacy of penicillin alternatives in HIV patients for latent infection or infection of unknown duration is poorly understood. However, some evidence has been provided to show doxycycline effectiveness for latent syphilis treatment in the HIV population [20].

*Treponema pallidum* can be transmitted vertically from an infected mother to the fetus during pregnancy or childbirth. This increases the risk and prevalence of congenital syphilis in patients of childbearing age, like the patient presented in this case. Indeed, the incidence of congenital syphilis in the United States has steadily increased since 2013 [1,6]. Appropriate screening and timely treatment for syphilis in women of childbearing age can prevent complications from a congenital infection. While prophylaxis could be helpful in patients with high-risk sexual behavior, further study is needed to establish practical interventions for this vulnerable population without contributing to antibiotic resistance. Prior studies involving HIV-positive men who have sex with men have shown benefits with 100 mg daily doxycycline prophylaxis and postexposure 200 mg doxycycline prophylaxis given 24 hours after a sexual encounter [1]; however, doxycycline exposure is not recommended at any stage of pregnancy [6], and thus would be a poor option for women of childbearing age. Further research should be conducted to develop prophylactic regimens for women of childbearing age.

## Conclusions

This report illustrates a case of syphilis of unknown duration with cervical lymphadenopathy and systemic symptoms suggestive of lymphoma. Clinicians should have a low threshold to test for syphilis in patients with unexplained lymphadenopathy, even without a typical rash or genital lesion. In this case, HIV and hepatitis C may have contributed to the patient's inability to mount an effective immune response to the syphilis infection. Patients with HIV and other immunocompromised states should be screened for sexually transmitted infections, as they are at increased risk for complications. This case emphasizes investigating patient history, including sexual history, throughout hospitalization. Heterosexual women of childbearing age are vulnerable to syphilis in the United States, particularly with co-existing drug use or HIV infection. Treatment of syphilis is effective but may require multiple doses for latent infection. Difficulties with transportation, relapse, healthcare costs, or lack of insurance increase the risk of similar patients being lost to follow-up, increasing the risk of non-remission, reinfection, and transmission. Further advancement in vaccine or prophylaxis methods in this population could mitigate rising primary and congenital syphilis rates.
